# Integrated network pharmacology, molecular docking and experimental validation to explore the mechanism of Dingji Fumai Decoction against LQTS

**DOI:** 10.1038/s41598-025-06515-7

**Published:** 2025-07-02

**Authors:** Xiaoyan Huang, Yanghong Jin, Jiangfang Lian

**Affiliations:** 1 Ningbo Institute of Innovation for Combined Medicine and Engineering, Ningbo Medical Center Lihuili Hospital, Ningbo, 315100 Zhejiang China; 2 Department of Cardiology, Ningbo Medical Center Lihuili Hospital, Ningbo, 315100 Zhejiang China

**Keywords:** Dingji Fumai Decoction, Long QT syndrome, Bioactive compounds, Network pharmacology, Molecular docking, Drug discovery, Cardiology

## Abstract

Dingji Fumai Decoction (DFD), a traditional herbal concoction, is commonly utilized in therapeutic practice to treat ventricular arrhythmia. However, research into the bioactive components and underlying processes of DFD in Long QT syndrome (LQTS) remains limited. All DFD compounds were gathered from the TCMSP, ETCM, and HERB databases, and the targets of active compounds were investigated using SwissTargetPrediction. The LQTS targets were obtained/screened from the DisGeNET, OMIM, and Malacard databases. The herb-compound-target-disease (H-C-T-D) and PPI networks were built using STRING and analyzed with CytoNCA based on the data obtained earlier. Meanwhile, VarElect was used to determine the relationship between targets and illnesses. The R program is used to enrich Gene Ontology (GO) terminology as well as the Kyoto Encyclopedia of Genes and Genomes (KEGG) pathways. The binding ability of DFD and hub genes was examined using molecular docking, followed by experimental validation of the key findings. A total of 664 DFD and 240 LQTS targets were gathered, with 21 common targets found. The H-C-T-D network demonstrated the links between DFD, active molecules, targets, and LQTS. The PPI network revealed the major targets: KCNH2, HSP90AA1, SCN5A, and CACNA2D1. Further systematic investigation revealed DFD’s potential mechanism for protecting against LQTS. Furthermore, molecular docking revealed the interactions of active drugs and targets. Finally, we discovered that DFD enhanced the levels of KCNH2, HSP90AA1, and CACNA2D1 while decreasing the level of SCN5A. DFD has the potential to cure LQTS through a complex method that involves interactions between active chemicals and targets. This study enhances our understanding of the molecular mechanisms underlying DFD’s effects on LQTS.

**Supplementary Information:**

The online version contains supplementary material available at 10.1038/s41598-025-06515-7.

## Introduction

LQTS is distinguished by delayed ventricular and atrial repolarization as well as prolonged QT interval on an electrocardiogram (ECG)^1^. These would have significantly raised the likelihood of the possibly deadly tachyarrhythmia Torsades de Pointes^2^. The majority of the genetic variants that cause congenital LQTS have been found, including KCNQ1 (LQT1), KCNH2 (LQT2), and SCN5A (LQT3)^3^. LQT1–LQT3 accounts for 90% of congenital LQTS, with LQT2 being the most common kind in China. KCNH2, also known as the human ether-à-go-go related gene (hERG), encodes the α-subunit of Kv11.1. Although techniques for preventing arrhythmia and sudden cardiac death have been established, they are often associated with side effects^4^. For instance, β-blockers are prescribed in LQTS. However, cardiovascular events and sudden deaths continue to occur. As a result, there is a significant unmet demand for lasting curative treatment for this condition.

Network pharmacology provides a novel paradigm to elucidate and map the complex interaction networks of Traditional Chinese Medicines (TCMs) in addressing multifactorial diseases. This methodology involves building networks that associate TCMs components with their respective molecular targets and subsequently connecting these targets to relevant disease pathways^5,6^. In the context of network pharmacology and TCMs, PPI networks are often used to explore the molecular mechanisms of TCMs formulations by identifying key proteins and pathways involved in their therapeutic effects. More TCMs standard prescriptions are being described at the molecular mechanism level and encouraged in clinical practice. Several TCMs, including Shensong Yangxin Capsule^7^ and Wenxin Keli^8^have been commercialized for the treatment of cardiovascular disorders. DFD is a traditional Chinese herbal formulation that has been widely used in clinical practice to treat ventricular arrhythmias, including conditions like premature ventricular contractions (PVCs), ventricular tachycardia (VT), and other related cardiac disorders. Its therapeutic effects are attributed to the synergistic actions of its multiple herbal components.

DFD contains *Chuanxiong Rhizoma* (Chuanxiong), *Jujubae Fructus* (Dazao), *Poria cocos* *(Schw.)* *Wolf* (Fuling), *Cinnamomi Ramulus* (Guizhi), *Silktree Albizia Bark* (Hehuanpi), *Os Draconis* (Longgu), *Crassostrea Gigas* (Muli), *Ziziphi Spinosae Semen* (Suanzaoren), *Radix Polygalae* (Yuanzhi), and *Licorice* (Gancao)^9^. A recent clinical investigation found that, after a 12-week follow-up, DFD significantly reduced the traditional Chinese medicine syndrome score and the number of arrhythmias when compared to metoprolol, with no adverse pharmacological effects and adequate patient medication adherence^10^. Furthermore, DFD’s antiarrhythmic processes are based on its antioxidant potential and class I antiarrhythmic capabilities, which involve inhibiting Nav1.5^11^. However, research on the active components of DFD and the basic mechanism for curing LQTS is currently limited. We explored the potential mechanism of DFD against LQTS using an integrated strategy combining network pharmacology, molecular docking, and experimental validation. First, we identified the active components of DFD and their potential targets, along with the key targets associated with LQTS. Subsequently, we conducted a systematic analysis of these candidate targets. Molecular docking was then employed to assess the binding interactions between DFD’s bioactive compounds and critical target proteins. Finally, the key findings were validated through molecular experiments. This study provides insights into the bioactive components of DFD and its underlying molecular mechanisms in the treatment of LQTS.

## Results

### Compounds screening and targets fishing

**Fig. 1:**
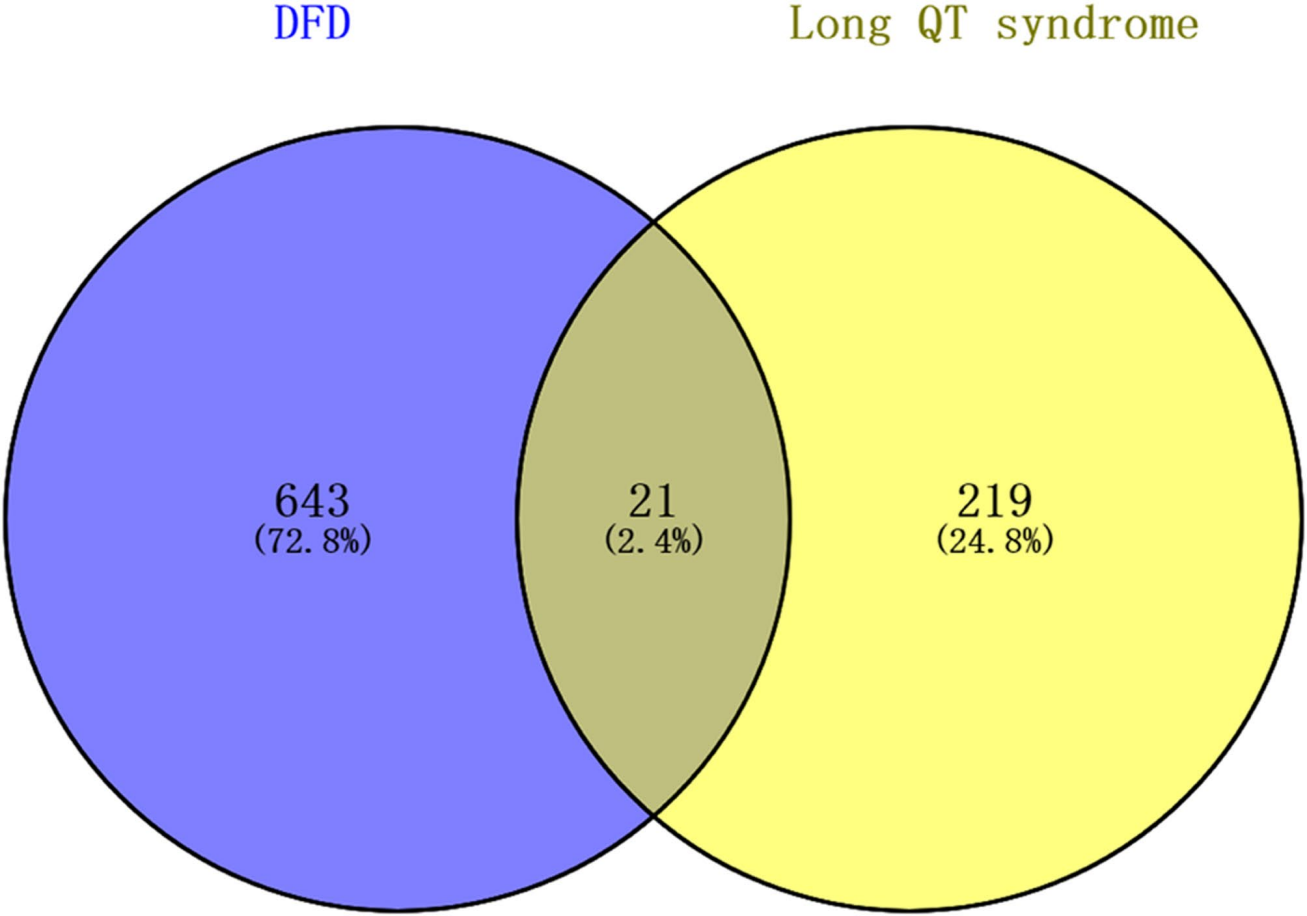
Venn diagram summarizing differential targets of DFD and LQTS.

**Fig. 2:**
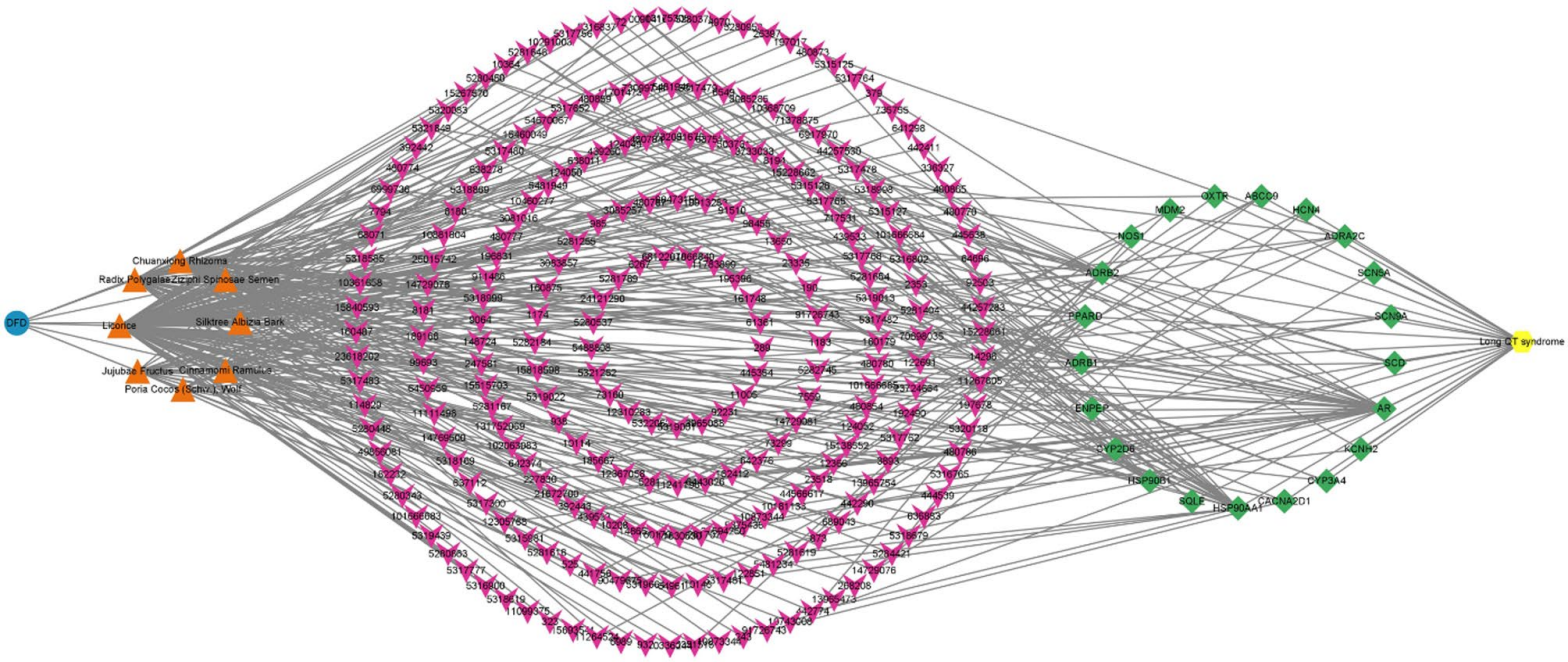
H-C-T-D network of DFD.

The TCMSP, ETCM, and HERB databases were examined for DFD compounds. We identified 141, 44, 31, 10, 14, 6, 14, and 33 chemicals in *Licorice*, *Chuanxiong Rhizoma*, *Jujubae Fructus*, *Poria cocos (Schw.) Wolf*, *Cinnamomi Ramulus*, *Silktree Albizia Bark*, *Ziziphi Spinosae Semen*, and *Radix Polygalae*. Later, the structures of each component were obtained from PubChem. After assessing each compound’s ADME with SwissADME, a total of 250 active compounds were verified, all of which had significant gastrointestinal absorption (GA) and drug-likeness (DL) potential. Supplementary Table 1 contains screening-related details. The bioactive compounds were used to predict possible targets using SwissTargetPrediction. After removing duplicates, a total of 664 targets were obtained. Following the removal of duplicates, OMIM, Malacards, and DisGeNET yielded a total of 240 identified therapeutic targets for LQTS. We discovered 21 shared targets between DFD and LQTS (Fig. 1). Using the data acquired above, Cytoscape was used to develop the H-C-T-D network (Fig. 2).

### Identification of hub genes

**Fig. 3:**
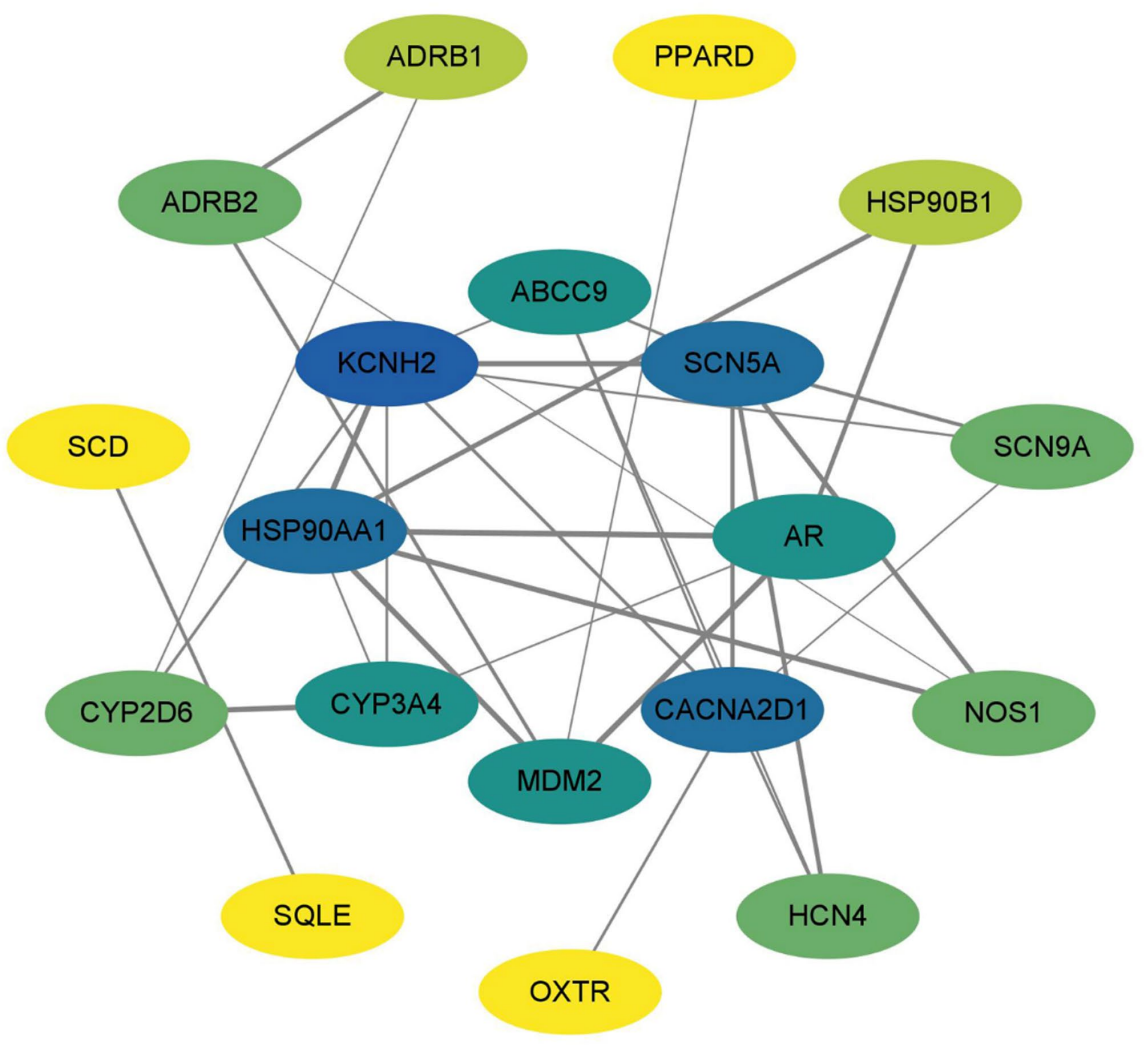
PPI network of potential targets of DFD against LQTS. Oval nodes on the inner circle indicate hub targets.

In order to establish the PPI network, all 21 common targets were uploaded to STRING. The results were then imported into Cytoscape, where the CytoNCA plugin was used to calculate the degree value of each gene and reconstruct the PPI network (Fig. 3). Following CytoNCA screening, hub targets and their designations and topological properties are shown in Table 1. The network’s high-degree protein targets may be responsible for DFD’s critical therapeutic benefits on LQTS. KCNH2, HSP90AA1, SCN5A, and CACNA2D1 are the most important of these targets. We used VarElect to assess 21 frequent targets and found a direct association between them and LQTS (Table 2).

**Table 1:** Designations and topological parameters of hub genes in the PPI network.

Gene symbol	Protein name	Degree	Betweenness centrality	Closeness centrality
KCNH2	Potassium voltage-gated channel subfamily H member 2	7	91.45	0.28
HSP90AA1	Heat shock protein HSP 90-alpha	6	70.34	0.27
SCN5A	Sodium channel protein type 5 subunit alpha	6	31.92	0.26
CACNA2D1	Voltage-dependent calcium channel subunit alpha-2/delta-1	6	36.54	0.25
CYP3A4	Cytochrome P450 3A4	4	15.52	0.26
AR	Androgen receptor	4	8.50	0.24
MDM2	E3 ubiquitin-protein ligase Mdm2	4	38.00	0.24
ABCC9	ATP-binding cassette sub-family C member 9	4	4.87	0.25
SCN9A	Sodium channel protein type 9 subunit alpha	3	0.00	0.24
NOS1	Nitric oxide synthase 1	3	25.72	0.26
CYP2D6	Cytochrome P450 2D6	3	18.50	0.24
ADRB2	Beta-2 adrenergic receptor	3	16.13	0.24
HCN4	Potassium/sodium hyperpolarization-activated cyclic nucleotide-gated channel 4	3	0.00	0.22
HSP90B1	Endoplasmin	2	0.00	0.22
ADRB1	Beta-1 adrenergic receptor	2	4.50	0.22
SQLE	Squalene monooxygenase	1	0.00	0.06
SCD	Stearoyl-CoA desaturase	1	0.00	0.06
PPARD	Peroxisome proliferator-activated receptor delta	1	0.00	0.20
OXTR	Oxytocin receptor	1	0.00	0.21

**Table 2:** The results of relevance analysis.

Symbol	Description	Score	Relationship
KCNH2	Potassium voltage-gated channel subfamily H member 2	316.09	Directely
SCN5A	Sodium voltage-gated channel alpha subunit 5	289.64	Directely
OXTR	Oxytocin receptor	76.54	Directely
CACNA2D1	Calcium voltage-gated channel auxiliary subunit alpha2delta 1	53.43	Directely
AR	Androgen receptor	49.86	Directely
HCN4	Hyperpolarization activated cyclic nucleotide gated potassium channel 4	47.77	Directely
ABCC9	ATP binding cassette subfamily C member 9	31.49	Directely
MDM2	MDM2 proto-oncogene	25.86	Directely
ADRB1	Adrenoceptor beta 1	24.32	Directely
SCN9A	Sodium Voltage-gated channel alpha subunit 9	23.37	Directely
CYP3A4	Cytochrome P450 family 3 subfamily A member 4	23.01	Directely
ADRB2	Adrenoceptor beta 2	21.53	Directely
CYP2D6	Cytochrome P450 family 2 subfamily D member 6	20.72	Directely
ADRA2C	Adrenoceptor Alpha 2 C	18.39	Directely
HSP90AA1	Heat shock protein 90 alpha family class A member 1	16.99	Directely
NOS1	Nitric oxide synthase 1	16.26	Directely
HSP90B1	Heat shock protein 90 beta family member 1	12.15	Directely
ENPEP	Glutamyl aminopeptidase	9.88	Directely
PPARD	Peroxisome proliferator activated receptor delta	6.20	Directely
SCD	Stearoyl-CoA desaturase	4.87	Directely
SQLE	Squalene epoxidase	3.21	Directely

### Functional enrichment analysis

**Fig. 4:**
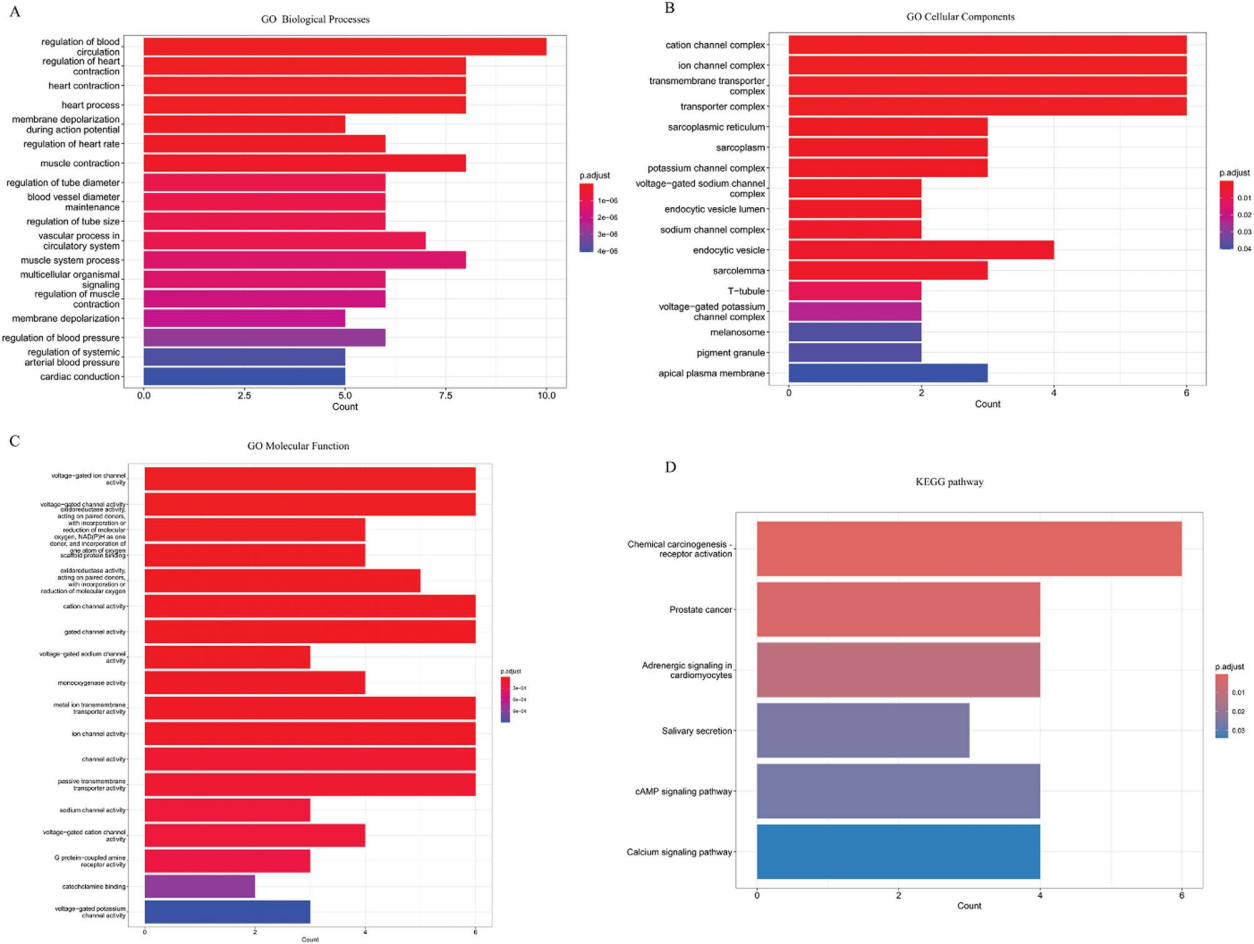
The functional enrichment analysis. (**A**) GO biological processes. (**B**) GO cellular components. (**C**) GO molecular function. (**D**) KEGG pathway.

We analyzed the biological features of probable DFD targets on LQTS using GO Biological Processes (BP), GO Molecular Function (MF), GO Cellular Components (CC), and KEGG pathway analysis. The data were shown using R’s ggplot tool. Figure 4 shows the most significant (*P* < 0.05) gene ontology categories. Common objectives in BP include regulation of blood circulation, regulation of heart contraction, heart contraction, heart process, membrane depolarization during action potential, regulation of heart rate, etc. And CC analysis showed that the common targets were cation channel complex, ion channel complex, transmembrane transporter complex, transporter complex, sarcoplasmic reticulum, sarcoplasm, potassium channel complex, etc. Numerous GO MF were involved in voltage-gated ion channel activity, voltage-gated channel activity, cation channel activity, gated channel activity, voltage-gated sodium channel activity, ion channel activity, channel activity, passive transmembrane transporter activity, sodium channel activity, etc.

We performed KEGG pathway analysis of these target genes to better understand the underlying processes involved in DFD’s cardioprotective effects on LQTS. Figure 4D depicts the most important KEGG pathway among the common targets. These shared targets were particularly abundant in pathways in chemical carcinogenesis—receptor activation, prostate cancer, adrenergic signaling in carcinogenesis—receptor activation, prostate cancer, adrenergic signaling in cardiomyocytes, salivary secretion, the cAMP signaling pathway, and the calcium signaling pathway.

### Molecular docking

**Fig. 5:**
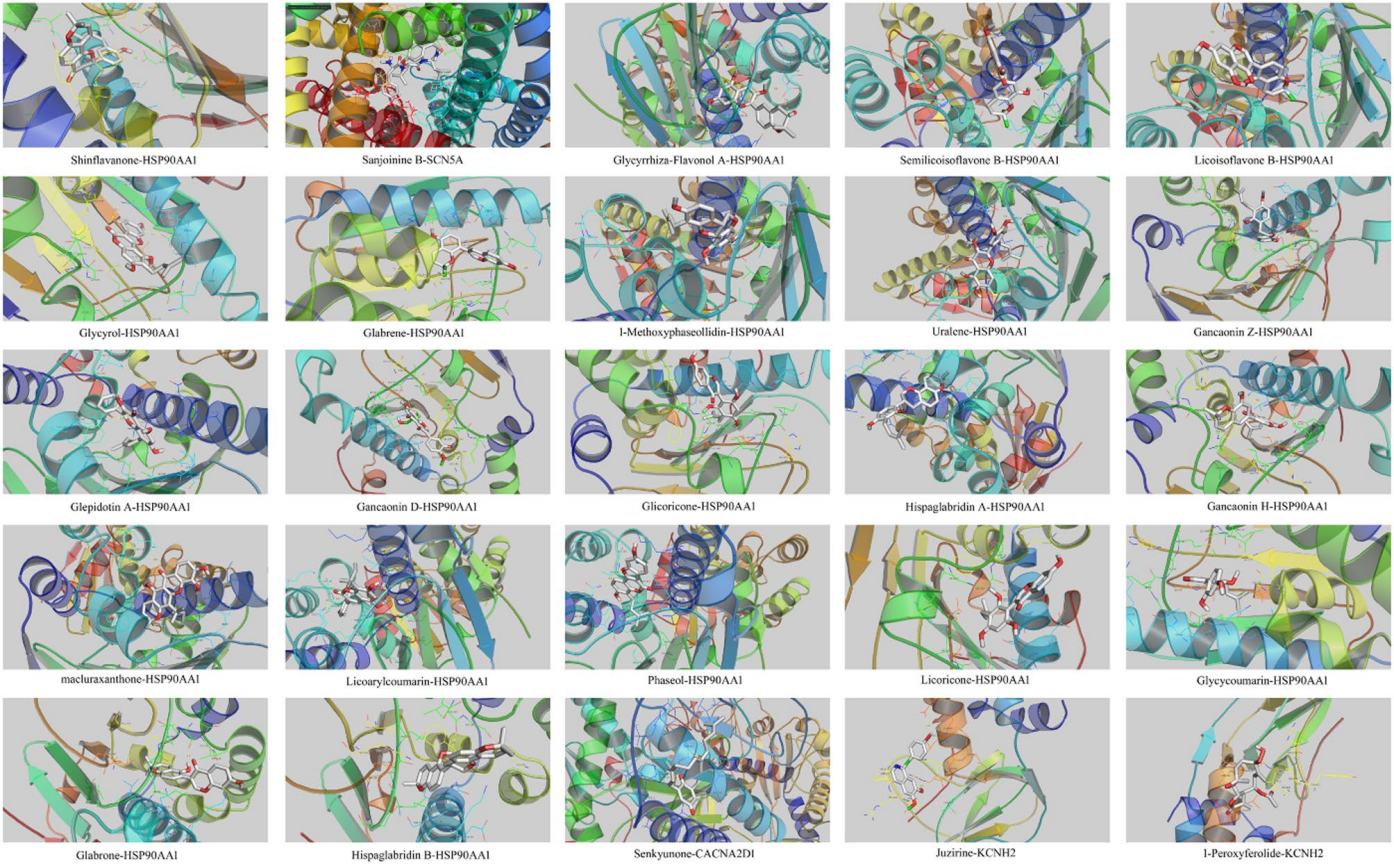
The binding energy molecular docking models.

Molecular docking was performed to assess the binding interactions and compute the binding energies between the DFD compounds and the four crucial targets linked to LQTS: KCNH2, HSP90AA1, SCN5A, and CACNA2D1. Table 3 shows docking information for components and hub targets. Results indicate that components in DFD interact with hub targets against LQTS, with lower binding energy leading to more stable docking modules. Shinflavanone formed binding energy of − 10.3 kcal/mol with HSP90AA1. Sanjoinine B has a binding energy of − 9.5 kcal/mol with SCN5A. Glycyrrhiza-Flavonol A has a binding energy of − 9.1 kcal/mol to HSP90AA1. The binding energy docking modules are depicted in Fig. 5. We employed the following target-inhibitor pairs as positive controls: KCNH2 (E-4031), SCN5A (Tetrodotoxin), HSP90AA1 (Tanespimycin), and CACNA2D1 (Gabapentin), with the corresponding binding energy profiles illustrated in Fig. 1S.

**Table 3:** The related information consists of components docked with key targets.

Compounds	Target genes	PDB-ID	Binding energy/kcal mol^− 1^
Shinflavanone	HSP90AA1	2YEI	− 10.3
Sanjoinine B	SCN5A	8F6P	− 9.5
Glycyrrhiza-Flavonol A	HSP90AA1	4L93	− 9.1
Semilicoisoflavone B	HSP90AA1	2XAB	− 8.6
Licoisoflavone B	HSP90AA1	2XAB	− 8.5
Glycyrol	HSP90AA1	4YKR	− 8.4
Glabrene	HSP90AA1	4YKR	− 8.2
1-Methoxyphaseollidin	HSP90AA1	4L93	− 8.2
Uralene	HSP90AA1	4L93	− 8.2
Gancaonin Z	HSP90AA1	4YKR	− 8.1
Glepidotin A	HSP90AA1	4L93	− 8.0
Gancaonin D	HSP90AA1	4YKR	− 7.8
Glicoricone	HSP90AA1	4YKR	− 7.8
Hispaglabridin A	HSP90AA1	2XAB	− 7.8
Gancaonin H	HSP90AA1	4YKR	− 7.7
macluraxanthone	HSP90AA1	4L93	− 7.7
Licoarylcoumarin	HSP90AA1	4L93	− 7.7
Phaseol	HSP90AA1	4L93	− 7.7
Licoricone	HSP90AA1	4YKR	− 7.6
Glycycoumarin	HSP90AA1	4YKR	− 7.5
Glabrone	HSP90AA1	4YKR	− 7.5
Hispaglabridin B	HSP90AA1	4YKR	− 7.2
Senkyunone	CACNA2D1	8IF4	− 5.0
Juzirine	KCNH2	6SYG	− 4.7
1-Peroxyferolide	KCNH2	6SYG	− 3.1

### Experimental validation

**Fig. 6:**
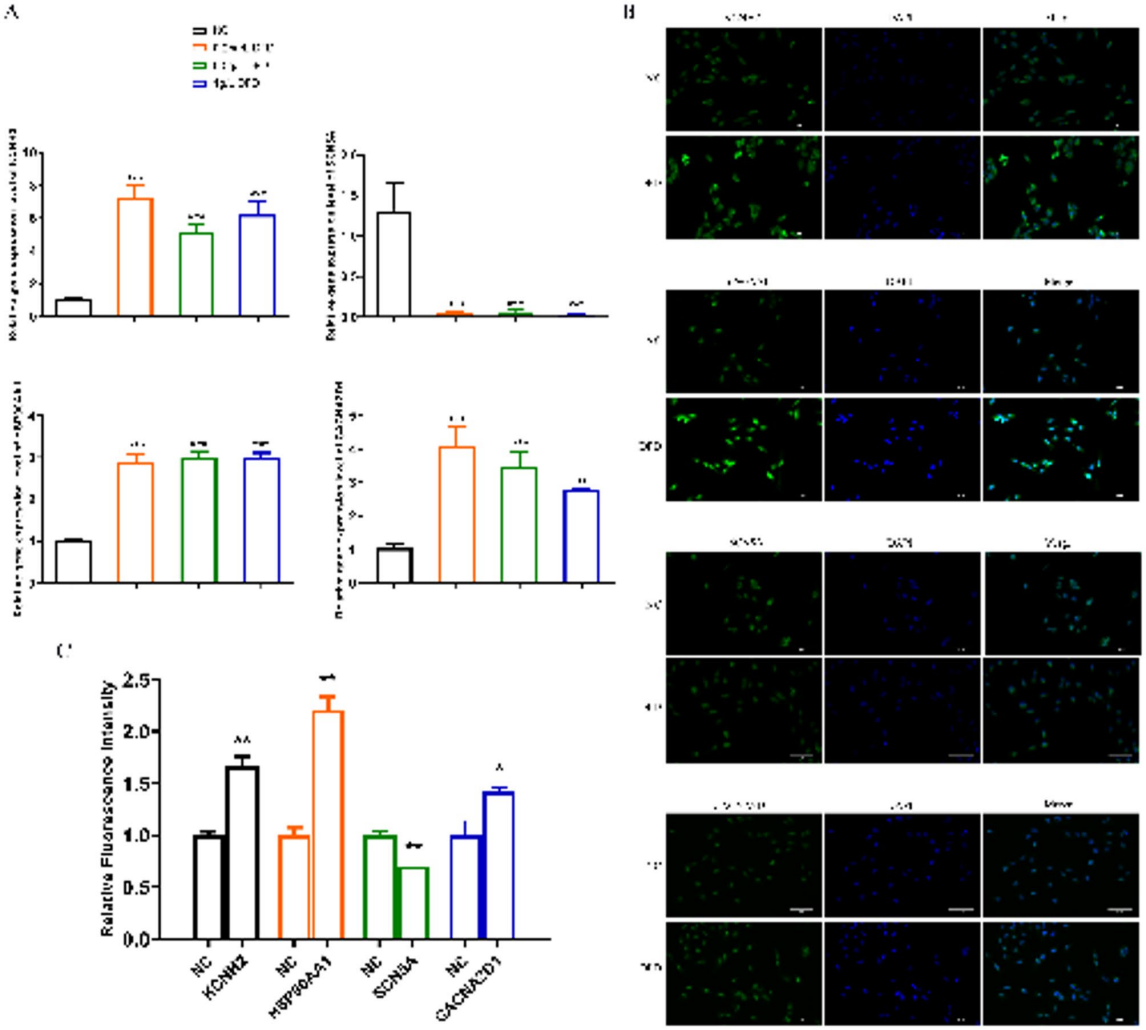
Experimental validation. (**A**) Gene expression levels of KCNH2, HSP90AA1, SCN5A, and CACNA2D1 in AC16 cells before and after DFD treatment. (**B**) Immunofluorescence staining of KCNH2, HSP90AA1, SCN5A, and CACNA2D1 expression in AC16 cells treated with DFD. The green fluorescence represents the location of the KCNH2, HSP90AA1, SCN5A, and CACNA2D1 proteins. The blue fluorescence represents the nucleus. (**C**) Relative fluorescence intensity of KCNH2, HSP90AA1, SCN5A, and CACNA2D1. Error bars represent the mean ± standard error of three independent experiments. *p < 0.05 compared with the control group, **p < 0.01 compared with the control group, and ***p < 0.001 compared with the control group.

First, we investigated the effect of DFD on the mRNA expression of hub targets KCNH2, HSP90AA1, SCN5A, and CACNA2D1 in AC16 cells. When 0.25, 0.5, and 1 g/L DFD were administered separately, mRNA levels of KCNH2, HSP90AA1, and CACNA2D1 increased significantly while SCN5A dropped compared with the control group, without concentration dependence (Fig. 6A). Then we investigated the effect of DFD on the protein expression of hub targets KCNH2, HSP90AA1, SCN5A, and CACNA2D1 with immunofluorescence staining. The green fluorescence represents the location of the hub proteins. The blue fluorescence represents the nucleus. Interestingly, we discovered that when 0.5 g/L DFD was administered, protein levels of KCNH2, HSP90AA1, and CACNA2D1 increased dramatically whereas SCN5A dropped compared with the control group (Fig. 6B,C).

## Discussion

Long-term clinical studies have shown that DFD is an effective herb combo for antiarrhythmia. Liang B et al.^10^ conducted a Real-World trial with over 160 patients who experienced premature ventricular contractions to assess the safety and efficacy of DFD for VA, and the results showed that DFD combined with metoprolol was more effective and safer than placebo combined with metoprolol. Furthermore, one mechanism of DFD in treating atrial fibrillation (AF) has been the suppression of Nav1.5, which has provided a supplement therapeutic method of TCMs for AF^11^. This study carefully examined the bioactive components and molecular processes of DFD for treating LQTS.

To comprehensively understand the therapeutic potential of DFD, a systematic analysis of its bioactive compounds and their corresponding targets was conducted. After evaluating the relevant datasets, a total of 250 bioactive compounds, 664 putative DFD targets, and 240 therapy-related LQTS targets were identified. Then we built the H-C-T-D network, which demonstrated the highly complicated relationships between herbs, targets, and diseases. This initially revealed the probable mechanism of DFD against LQTS and identified 21 candidate targets. VarElect was used to examine all possible genes. All 21 hub genes appeared to be directly associated to LQTS treatment, with KCNH2, HSP90AA1, SCN5A, and CACNA2D1 having the highest correlation values. KCNH2 has recently emerged as a hot gene in the research of VA, possibly mediating the quickly activating component of the delayed rectifying potassium current in the heart. Previous studies revealed that pathogenic mutations in KCNH2 encoding could cause LQTS, and the KCNH2 gene mutation rate was greater than 85% among LQTS patients^12,13^. SCN5A has a critical role in arrhythmic risk and cardiac electrical conduction. A study that prevented Nav1.5 downregulation offered a useful treatment to lessen arrhythmia^14^. Quinidine and mexiletine together were shown in another trial to decrease arrhythmia in patients with SCN5A gene mutations^15^. HSP90AA1, a molecular chaperone belonging to the HSP90 family, has helped with the maturation of different proteins as well as the assembly of intracellular molecules and protein folding^16^. HSPs help maintain ion channel and cytoskeletal stability, reducing atrial remodeling caused by AF^17^. It is encouraging to observe that HSP90 has been demonstrated to influence a range of cardiac arrhythmias, including long QT interval syndrome, through its modulation of proteins associated with cellular ion channels^18^. Overexpression of the CACNA2D1 gene affects the expression of the α2δ-1 calcium ion channel protein, which affects calcium ion transport and concentration inside and outside the cell^19,20^. Exercise-induced downregulation of CACNA2D1 and a decrease in Ca^2+^ concentration both diastolized vascular smooth muscle and attenuated the cardiac damage caused by hypertension in mice^21^. While not a primary cause of congenital LQTS, CACNA2D1 may modulate calcium handling and indirectly affect repolarization.

To further understand the biological significance of these hub genes, a functional enrichment analysis was conducted. This analysis aimed to explore the potential roles and pathways associated with the identified genes, providing deeper insights into their functional relevance within the biological context. The GO analysis results clarified the regulation of heart contraction, blood circulation, heart process, transmembrane transporter complex, cation channel complex, ion channel complex, voltage-gated ion channel activity, gated channel activity, and voltage-gated sodium channel activity, revealing possible mechanisms of DFD against LQTS. The KEGG pathway study has indicated that DFD has played a role in adrenergic signaling in cardiomyocytes, as well as the cAMP and calcium signaling pathways. Adrenergic signaling, driven by catecholamines, modulates heart rate, myocardial contractility, and relaxation via β-adrenergic receptors (β-ARs). Excessive activation of β-AR signaling may contribute to the development of arrhythmias, cardiac hypertrophy, and apoptosis^22,23^. The cAMP signaling pathway plays a crucial role in regulating essential physiological processes, including gene transcription, calcium homeostasis, and muscle contraction^24^. Berisha et al.^25^ explored the role of cAMP signaling dynamics at ryanodine receptors (RyR2) in the pathogenesis of cardiac arrhythmias, particularly in the context of heart failure and hypertrophy. Another important pathway associated with the development of LQTS that DFD involves is the calcium signaling pathway, which is crucial for the regulation of muscle contraction, calcium ion binding, transport, and cell metabolism^26^. Moreover, extensive population-based GWAS studies has identified several loci that encode Ca^2+^-signaling proteins, which are associated with longer QT durations^27^. Although most of the recognized LQTS-related genes encode proteins that regulate the movement of Na^+^ and K^+^ across the sarcolemma, there is growing evidence that Ca^2+^ fluxes and intracellular Ca^2+^ signaling play a role in prolonged cardiac repolarization and LQTS^28^. The involvement of DFD in adrenergic signaling, cAMP signaling, and calcium signaling pathways highlights its potential as a multi-target therapy for LQTS and other cardiovascular disorders. By modulating these pathways, DFD could address the underlying electrophysiological and structural abnormalities in LQTS, offering a promising complementary approach to conventional treatments.

This potential is further supported by molecular docking studies, which provide insights into the interactions between DFD and key targets. The docking results revealed the strongest binding forces between Shinflavanone and HSP90AA1 (− 10.3 kcal/mol), Sanjoinine B and SCN5A (− 9.5 kcal/mmol), and Glycyrrhiza-Flavonol A and HSP90AA1 (− 9.1 kcal/mmol). It suggests that the core active components can spontaneously and stably join with the core target proteins (KCNH2, HSP90AA1, SCN5A, and CACNA2D1) and play an important role in the therapy of LQTS. The molecular results of our investigation indicated that DFD and active substances enhanced the levels of KCNH2, HSP90AA1, and CACNA2D1 while decreasing SCN5A expression. Taken together, the findings indicated that DFD reduced LQTS by regulating KCNH2, HSP90AA1, CACNA2D1, and SCN5A.

DFD, a traditional Chinese decoction, treats LQTS using several components and targets, implying that the underlying mechanisms are complicated, as outlined in our current work. Although we have identified some of the mechanisms, there is still much more to discover. Undoubtedly, some targets involving either DFD or LQTS may be overlooked and neglected, which is a common and unavoidable problem in network pharmacology. Additionally, so many components are prepared together that more research is required to determine whether any new compounds are produced. These new compounds could contribute to the therapeutic effects but are often overlooked in studies. Metabolomics and proteomics techniques can help identify new compounds formed during preparation or metabolism and their biological effects. Finally, While network pharmacology has provided valuable insights into the mechanisms of DFD in treating LQTS, the complexity of both the formulation and the disease means that much remains to be discovered. Addressing the limitations of current approaches and leveraging advanced technologies will be key to unlocking the full potential of DFD and other TCM formulations.

## Methods

### Acquisition of bioactive compounds and potential targets of DFD

Initially, the bioactive chemicals identified in DFD were gathered from the TCMSP, ETCM, and the HERB databases. The structures of each drug were taken from PubChem^29^. Next, each compound’s absorption, distribution, metabolism, and excretion (ADME) were determined using SwissADME with GA and DL. Bioactive chemicals are those that have a high possibility of containing both GA and DL^9,30^. Finally, bioactive compounds were chosen for potential target screening with SwissTargetPrediction^31^.

### Collection of gene targets for LQTS

Targets for LQTS were gathered from the DisGeNET database^32, ^OMIM^33, ^and MalaCards^34^. Using Venny 2.1, the intersection of DFD and LQTS targets was used to determine DFD’s therapeutic targets against LQTS.

### Construction of herb-compound-target-disease network

Using the acquired data, we created the H-C-T-D network in Cytoscape 3.9.0 software to demonstrate the relationship between bioactive chemicals and possible targets. The targets were used to investigate further protein interactions and conduct functional enrichment analyses.

### Protein–protein interaction exploration

PPI is utilized to clarify protein interactions and to assist with the functional properties of hypothetical protein complexes^35^. In this study, shared targets were used to explore PPI using STRING, a web database that allows for online PPI analysis. The creature was labeled “homo sapiens,” and the unconnected nodes were hidden. We used a confidence score threshold of 0.400 for selecting PPIs from the STRING database. The findings were then analyzed in Cytoscape, with the CytoNCA plugin used to screen for hub genes^36^. Furthermore, to more precisely survey the hub gene, we used the VarElect dataset, an online tool that may rate the likelihood of genes being associated with specific illnesses^37^ for the relevance analysis between the gene symbol of shared targets and LQTS.

### Functional annotation of key targets

In this work, functional enrichment analysis focused on the GO resource and the KEGG dataset^38–40^ both of which contributed to gene functional interpretation and discovery. The analysis considered GO terms and KEGG pathways with P-value below 0.05 as statistically significant. The findings were plotted using R’s ggplot2 package^41^.

### Molecular docking

Hub proteins based on PPI communications were prepared for docking. The crystal structures of DFD bioactive drug potential protein targets were obtained from the RCSB Protein Data Bank (https://www.rcsb.org/) and altered using Autodock 4.2 software, which allowed for ligand and water removal, hydrogen addition, and amino acid optimization and patching. ChemBioDraw 3D was used to create the most energy-efficient 3D chemical structures. The output was saved in MOL.2 format. The molecular docking was then carried out utilizing Autodock Vina. The binding energy between the receptor and the ligand was below 0 kcal/mol, indicating a high affinity for docking^42^.

### Experimental validation

#### Cell culture and drug exposure

The human cardiomyocyte AC16 cell line was provided by Starfish (Hycyte). The cells were cultured in DMEM/F12 (Cat #: 10-092-CVRC, CORNING) supplemented with 10% FBS (Cat #: FBP-C520, Hysigen) at 37 °C with 5% CO_2_. DFD was made into freeze-dried powder.

#### Quantitative real-time reverse transcription PCR

Total RNA was extracted using the Total RNA Kit (Cat #: R6834, Omega) according to the manufacturer’s instructions. The extracted RNA from each sample was reverse transcribed using ABScript III RT Master Mix for qPCR with gDNA Remover (Cat #: RK20429, ABclonal) according to the manufacturer’s instructions. Real-time PCR was performed in triplicate utilizing the produced cDNA and 2×SuperFast Universal SYBR Master Mix (Cat #: CW3888H, CWBIO). The primer sequences used are listed in Table 4. The samples were then cycled in an Applied Biosystems 7500 RT-PCR machine using the following settings: 30 s at 95 °C, followed by 45 cycles of 10 s at 95 °C and 30 s at 60 °C. The 2^−ΔΔCt^ approach was used to calculate relative quantification and normalize it to GAPDH levels.

**Table 4:** Pairs of forward-reverse primers.

S. No	Gene	Primer sequence	
		Forward primer	Reverse primer
1	GAPDH	GTTCGTCATGGGTGTGAACC	GCATGGACTGTGGTCATGAGT
2	KCNH2	TTTGAGGGCCAGAGCCGTAA	TGCAGGAAGTCGCAGGTG
3	Hsp90AA1	CGTTTCTGAGAAGCAGGGCA	GGGAATGCAGAGACGTGGAA
4	SCN5A	GTCTGCCCACCCTGCTCTCTG	ACCAGCTGCTTCCCTGAATC
5	CACNA2D1	ACGCAGCAGTCCATATTCCTA	GCCACAATAATGAAGGGTCTTCC

#### Immunofluorescence staining

AC16 cells were fixated in 4% PFA, permeabilized in 0.3% Triton X-100, then blocked with 5% BSA. The cells were then treated overnight with primary antibodies. The main antibodies utilized were SCN5A antibody (Cat #: ASC-013; 1/200; Alomone Labs), KCNH2 rabbit pAb (Cat #: A2968; 1/100; ABclonal), HSP90AA1 rabbit pAb (Cat #: A0365; 1/100; ABclonal), and CACNA2D1 antibody (Cat #: DF8510; 1/200; Affinity). Secondary antibodies, such as ABflo^®^ 488-conjugated Goat anti-Rabbit IgG (H + L) (Cat #: AS073; 1/200; ABclonal), were applied for 1 h and counterstained with DAPI. The observation was carried out in a darkroom with a fluorescence microscope. Image processing and quantification were done with ImageJ software.

### Statistical analysis

GraphPad Prism 8.0 software (GraphPad Software, Inc.) was used to perform statistical analysis. Differences between two groups were analyzed using an unpaired Student’s t-test. All experiments were performed at least in triplicate and data are presented as the mean ± SD. Results were judged statistically significant at *p* < 0.05.

## Electronic supplementary material

Below is the link to the electronic supplementary material.

Supplementary Material 1

Supplementary Material 2

## Data Availability

The datasets analyzed during the current study are available from the corresponding author on reasonable request.
