# Supplementary material for: Integrated network pharmacology, molecular docking and experimental validation to explore the mechanism of Dingji Fumai Decoction against LQTS
Source: Sci Rep. 2025 Jul 2;15:23037. doi: 10.1038/s41598-025-06515-7 (PMC12215522; doi:10.1038/s41598-025-06515-7)
Supplement: Supplementary file 1 — Supplementary Material 1 [file 41598_2025_6515_MOESM1_ESM.docx]

### Table 1S . Details of qualified compounds in Dingji Fumai Decoction.

| PUBCHEM CID | ingredients | SwissADME results | | | | | |  |
| --- | --- | --- | --- | --- | --- | --- | --- | --- |
|  |  | Gatrointestinal absorption(GA) | druglikeness (DL) | | | | |  |
|  |  |  | lipinski | ghose | veber | egan | muegge | |
| 689043 | Caffeic Acid | high | √ | √ | √ | √ |  | |
| 12366 | Ethyl Palmitate | high | √ |  |  |  |  | |
| 1183 | Vanillin | high | √ |  | √ | √ |  | |
| 1174 | Uracil | high | √ |  | √ | √ |  | |
| 61361 | Butylphthalide | high | √ | √ | √ | √ |  | |
| 10208 | Chrysophanic Acid | high | √ | √ | √ | √ | √ | |
| 10873344 | Wallichilide | high | √ | √ | √ | √ | √ | |
| 14296 | Chuanxiongzine | high | √ |  |  |  |  | |
| 642376 | 3-Butylidenephthalide | high | √ | √ | √ | √ |  | |
| 3085257 | Senkyunolide | high | √ | √ | √ | √ |  | |
| 92231 | Spathulenol | high | √ | √ | √ | √ |  | |
| 12367058 | Sedanonic Acid | high | √ | √ | √ | √ | √ | |
| 5315981 | Cnidium Lactone | high | √ | √ | √ | √ |  | |
| 70698035 | (Z,Z')-Diligustilide | high | √ | √ | √ | √ | √ | |
| 26397 | Ethyl Heptadecanoate | high | √ |  |  |  |  | |
| 13650 | 3-Hydroxycoumarin | high | √ |  | √ | √ |  | |
| 5319022 | Ligustilide | high | √ | √ | √ | √ |  | |
| 68122670 | 2-Methoxy-4-(3-Methoxy-1-Propenyl)-Phenol | high | √ | √ | √ | √ |  | |
| 8181 | Methyl Palmitate | high | √ |  |  | √ |  | |
| 5284421 | Methyl Linoleate | high | √ |  |  |  |  | |
| 23518 | Methyl Pentadecanoate | high | √ | √ |  | √ |  | |
| 7559 | Methyl Phenylacetate | high | √ |  | √ | √ |  | |
| 3083857 | Neocnidilide | high | √ | √ | √ | √ |  | |
| 445354 | Retinol | high | √ | √ | √ | √ |  | |
| 642374 | Senkyunolide C | high | √ | √ | √ | √ | √ | |
| 11264524 | Senkyunolide D | high | √ | √ | √ | √ | √ | |
| 11830530 | Senkyunolide E | high | √ | √ | √ | √ | √ | |
| 11241196 | Senkyunolide F | high | √ | √ | √ | √ | √ | |
| 10013283 | Senkyunolide G | high | √ | √ | √ | √ | √ | |
| 13965088 | Senkyunolide H | high | √ | √ | √ | √ | √ | |
| 24121290 | Senkyunolide J | high | √ | √ | √ | √ | √ | |
| 5321252 | Senkyunolide K | high | √ | √ | √ | √ | √ | |
| 13965754 | Senkyunolide L | high | √ | √ | √ | √ | √ | |
| 15138552 | Senkyunolide N | high | √ | √ | √ | √ | √ | |
| 91726743 | Senkyunone | high | √ |  | √ | √ |  | |
| [5282184](https://pubchem.ncbi.nlm.nih.gov/compound/5282184) | [Mandenol](https://old.tcmsp-e.com/molecule.php?qn=1494) | high | √ |  |  |  |  | |
| [161748](https://pubchem.ncbi.nlm.nih.gov/compound/161748) | [Myricanone](https://old.tcmsp-e.com/molecule.php?qn=2135) | high | √ | √ | √ | √ | √ | |
| [160179](https://pubchem.ncbi.nlm.nih.gov/compound/160179) | [Perlolyrine](https://old.tcmsp-e.com/molecule.php?qn=2140) | high | √ | √ | √ | √ | √ | |
| [91726743](https://pubchem.ncbi.nlm.nih.gov/compound/91726743) | [senkyunone](https://old.tcmsp-e.com/molecule.php?qn=2151) | high | √ |  | √ | √ |  | |
| [10873344](https://pubchem.ncbi.nlm.nih.gov/compound/10873344) | [wallichilide](https://old.tcmsp-e.com/molecule.php?qn=2157) | high | √ | √ | √ | √ | √ | |
| 5280450 | Linoleic Acid | high | √ |  |  |  |  | |
| 985 | Cetylic Acid | high | √ | √ |  | √ |  | |
| 11005 | Myristic Acid | high | √ | √ |  | √ |  | |
| 5281 | Stearic Acid | high | √ |  |  |  |  | |
| 525 | Malic Acid | high | √ |  | √ | √ |  | |
| 2353 | Berberine | high | √ | √ | √ | √ | √ | |
| 4970 | Protopine | high | √ | √ | √ | √ | √ | |
| 98455 | Stepharine | high | √ | √ | √ | √ | √ | |
| 938 | Nicotinic Acid | high | √ |  | √ | √ |  | |
| 6267 | Asparagine | high | √ |  | √ | √ |  | |
| 99693 | Skimmin | high | √ |  | √ | √ | √ | |
| 10146 | Nuciferine | high | √ | √ | √ | √ | √ | |
| 445638 | Palmitoleic Acid | high | √ | √ |  | √ |  | |
| 5282745 | 9-Hexadecenoic Acid | high | √ | √ |  | √ |  | |
| 160875 | Asimilobine | high | √ | √ | √ | √ | √ | |
| 289 | Catechol | high | √ |  | √ | √ |  | |
| 21672700 | Colubrinic Acid | high | √ |  | √ |  |  | |
| 441756 | [Spiradine A](https://old.tcmsp-e.com/molecule.php?qn=12940) | high | √ | √ | √ | √ | √ | |
| 5281707 | [coumestrol](https://old.tcmsp-e.com/molecule.php?qn=12976) | high | √ | √ | √ | √ | √ | |
| 6443026 | [Mauritine D](https://old.tcmsp-e.com/molecule.php?qn=12992) | high | √ |  |  | √ |  | |
| 90473155 | [malkangunin](https://old.tcmsp-e.com/molecule.php?qn=5360) | high | √ | √ | √ | √ | √ | |
| 6917970 | [Stepholidine](https://old.tcmsp-e.com/molecule.php?qn=627) | high | √ | √ | √ | √ | √ | |
| 5280537 | [Moupinamide](https://old.tcmsp-e.com/molecule.php?qn=8647) | high | √ | √ | √ | √ | √ | |
| 73160 | [(-)-catechin](https://old.tcmsp-e.com/molecule.php?qn=96) | high | √ | √ | √ | √ | √ | |
| 3893 | Lauric Acid | high | √ | √ | √ | √ | √ | |
| 379 | Caprylic Acid | high | √ |  | √ | √ |  | |
| 190 | Adenine | high | √ |  | √ | √ |  | |
| 247581 | Dehydroabietic Acid Methyl Ester | high | √ | √ | √ | √ |  | |
| 10368709 | 25-Hydroxy-3-Epidehydrotumulosic Acid | high | √ |  | √ | √ | √ | |
| 8180 | Undecanoic Acid | high | √ | √ | √ | √ |  | |
| 10743008 | [(2R)-2-[(3S,5R,10S,13R,14R,16R,17R)-3,16-dihydroxy-4,4,10,13,14-pentamethyl-2,3,5,6,12,15,16,17-octahydro-1H-cyclopenta[a]phenanthren-17-yl]-6-methylhept-5-enoic acid](https://old.tcmsp-e.com/molecule.php?qn=273) | high | √ |  | √ |  |  | |
| 10181133 | [Cerevisterol](https://old.tcmsp-e.com/molecule.php?qn=279) | high | √ |  | √ | √ |  | |
| 73299 | [hederagenin](https://old.tcmsp-e.com/molecule.php?qn=296) | high | √ |  | √ |  |  | |
| 5281255 | Isobavachalcone | high | √ | √ | √ | √ |  | |
| 68071 | (S)-5,7-Dihydroxy-2-Phenylchroman-4-One | high | √ | √ | √ | √ | √ | |
| 5317480 | Lupiwighteone | high | √ | √ | √ | √ | √ | |
| 5316900 | 3,3'-Dimethylquercetin | high | √ | √ | √ | √ | √ | |
| 5316802 | (E)-1-[2,4-Dihydroxy-3-(3-Methyl-2-Butenyl)Phenyl]-3-(4-Hydroxy-3-[3-Methyl-2-Butenyl)Phenyl]-2-Propen-1-One | high | √ |  | √ | √ |  | |
| 5280378 | Formononetin | high | √ | √ | √ | √ | √ | |
| 3733033 | Formononetin-7-Glucoside | high | √ | √ | √ |  | √ | |
| 5317478 | Gancaonin A | high | √ | √ | √ | √ | √ | |
| 5317479 | Gancaonin B | high | √ | √ | √ | √ | √ | |
| 6450959 | Gancaonin C | high | √ | √ | √ | √ | √ | |
| 5317481 | Gancaonin D | high | √ | √ | √ | √ | √ | |
| 480770 | Gancaonin E | high | √ | √ | √ | √ |  | |
| 5317482 | Gancaonin F | high | √ | √ | √ | √ | √ | |
| 480777 | Gancaonin I | high | √ | √ | √ | √ |  | |
| 5317483 | Gancaonin P-3'-Methylether | high | √ | √ | √ | √ | √ | |
| 101666683 | Gancaonin X | high | √ | √ | √ | √ | √ | |
| 90479675 | Glabrolide | high | √ |  | √ | √ |  | |
| 480786 | Glisoflavanone | high | √ | √ | √ | √ |  | |
| 5317756 | Glycycoumarin | high | √ | √ | √ | √ | √ | |
| 480787 | Glycyrin | high | √ | √ | √ | √ | √ | |
| 5320083 | Glycyrol | high | √ | √ | √ | √ | √ | |
| 10114 | Glycyrrhetinic Acid | high | √ |  | √ |  |  | |
| 12310283 | Glycyrrhetol | high | √ |  | √ |  |  | |
| 5317762 | Glycyrrhisoflavanone | high | √ | √ | √ | √ | √ | |
| 5317764 | Glycyrrhisoflavone | high | √ | √ | √ | √ | √ | |
| 5317765 | Glycyrrhiza-Flavonol A | high | √ | √ | √ | √ | √ | |
| 15818598 | Glyurallin A | high | √ | √ | √ | √ | √ | |
| 195396 | Glyuranolide | high | √ |  | √ | √ | √ | |
| 5317777 | Glyzaglabrin | high | √ | √ | √ | √ | √ | |
| 442774 | Hispaglabridin A | high | √ | √ | √ | √ |  | |
| 15228661 | Hispaglabridin B | high | √ | √ | √ | √ |  | |
| 480854 | 3-Hydroxyglabrol | high | √ | √ | √ | √ |  | |
| 124050 | Isoglycyrol | high | √ | √ | √ | √ | √ | |
| 5318585 | Isolicoflavonol | high | √ | √ | √ | √ | √ | |
| 638278 | Isoliquiritigenin | high | √ | √ | √ | √ | √ | |
| 5318619 | Isoononin | high | √ | √ | √ |  | √ | |
| 5318679 | Isotrifoliol | high | √ | √ | √ | √ | √ | |
| 131753069 | Kanzonol K | high | √ |  | √ | √ |  | |
| 503731 | Licocoumarone | high | √ | √ | √ | √ | √ | |
| 5319001 | Licofuranocoumarin | high | √ | √ | √ | √ | √ | |
| 5281789 | Licoisoflavone | high | √ | √ | √ | √ | √ | |
| 11111496 | Licoleafol | high | √ | √ | √ | √ | √ | |
| 122851 | Licopyranocoumarin | high | √ | √ | √ | √ | √ | |
| 480865 | Licoricidin | high | √ | √ | √ | √ |  | |
| 5319013 | Licoricone | high | √ | √ | √ | √ | √ | |
| 196831 | Licorisoflavan A | high | √ |  | √ | √ |  | |
| 114829 | Liquiritigenin | high | √ | √ | √ | √ | √ | |
| 5319439 | 3'-Methoxyglabridin | high | √ | √ | √ | √ | √ | |
| 5319664 | 4'-O-Methylglabridin | high | √ | √ | √ | √ | √ | |
| 5320118 | Neouralenol | high | √ | √ | √ | √ | √ | |
| 162412 | Phaseollinisoflavan | high | √ | √ | √ | √ | √ | |
| 73205 | Sigmoidin B | high | √ | √ | √ | √ | √ | |
| 5321849 | 5,6,7,8-Tetrahydro-2,4-Dimethylquinoline | high | √ | √ | √ | √ |  | |
| 185667 | 5,6,7,8-Tetrahydro-4-Methylquinoline | high | √ |  | √ | √ |  | |
| 5322052 | 2,4,4'-Trihydroxychalcone | high | √ | √ | √ | √ | √ | |
| 192490 | Uralene | high | √ | √ | √ | √ | √ | |
| 5315125 | Uralenin | high | √ | √ | √ | √ | √ | |
| 5315126 | Uralenol | high | √ | √ | √ | √ | √ | |
| 5315127 | Uralenol-3-Methylether | high | √ | √ | √ | √ | √ | |
| 11783899 | Uralstilbene | high | √ | √ | √ | √ |  | |
| 10881804 | Kanzonol B | high | √ | √ | √ | √ | √ | |
| 13965473 | 3',7-Dihydroxy-4',6-Dimethoxyisoflavone | high | √ | √ | √ | √ | √ | |
| 44257283 | Erythrinin C | high | √ | √ | √ | √ | √ | |
| 101666685 | Gancaonin Y | high | √ | √ | √ | √ | √ | |
| 101666684 | Gancaonin Z | high | √ | √ | √ | √ | √ | |
| 10361658 | Glicoricone | high | √ | √ | √ | √ | √ | |
| 5318869 | Kumatakenin | high | √ | √ | √ | √ | √ | |
| 11099375 | Licoagrochalcone A | high | √ | √ | √ | √ |  | |
| 636883 | Licoagroisoflavone | high | √ | √ | √ | √ | √ | |
| 10090416 | Licoarylcoumarin | high | √ | √ | √ | √ | √ | |
| 91510 | Maackiain | high | √ | √ | √ | √ | √ | |
| 44257530 | Phaseol | high | √ | √ | √ | √ | √ | |
| 5481948 | Semilicoisoflavone B | high | √ | √ | √ | √ | √ | |
| 14769500 | Xambioona | high | √ | √ | √ | √ | √ | |
| 5481234 | Licoisoflavone B | high | √ | √ | √ | √ | √ | |
| 336327 | [Medicarpin](https://old.tcmsp-e.com/molecule.php?qn=2565) | high | √ | √ | √ | √ | √ | |
| 5281654 | [isorhamnetin](https://old.tcmsp-e.com/molecule.php?qn=354) | high | √ | √ | √ | √ | √ | |
| 911486 | [7-Methoxy-2-methyl isoflavone](https://old.tcmsp-e.com/molecule.php?qn=3896) | high | √ | √ | √ | √ | √ | |
| 5280448 | [Calycosin](https://old.tcmsp-e.com/molecule.php?qn=417) | high | √ | √ | √ | √ | √ | |
| 5280863 | [kaempferol](https://old.tcmsp-e.com/molecule.php?qn=422) | high | √ | √ | √ | √ | √ | |
| 932 | [naringenin](https://old.tcmsp-e.com/molecule.php?qn=4328) | high | √ | √ | √ | √ | √ | |
| 197678 | [(2S)-2-[4-hydroxy-3-(3-methylbut-2-enyl)phenyl]-8,8-dimethyl-2,3-dihydropyrano[2,3-f]chromen-4-one](https://old.tcmsp-e.com/molecule.php?qn=4805) | high | √ | √ | √ | √ |  | |
| 10291003 | [euchrenone](https://old.tcmsp-e.com/molecule.php?qn=4806) | high | √ | √ | √ | √ | √ | |
| 480784 | [glyasperin B](https://old.tcmsp-e.com/molecule.php?qn=4808) | high | √ | √ | √ | √ | √ | |
| 392442 | [glyasperin F](https://old.tcmsp-e.com/molecule.php?qn=4810) | high | √ | √ | √ | √ | √ | |
| 480859 | [Glyasperin C](https://old.tcmsp-e.com/molecule.php?qn=4811) | high | √ | √ | √ | √ | √ | |
| 637112 | [(2S)-6-(2,4-dihydroxyphenyl)-2-(2-hydroxypropan-2-yl)-4-methoxy-2,3-dihydrofuro[3,2-g]chromen-7-one](https://old.tcmsp-e.com/molecule.php?qn=4824) | high | √ | √ | √ | √ | √ | |
| 5281619 | [Glepidotin A](https://old.tcmsp-e.com/molecule.php?qn=4828) | high | √ | √ | √ | √ | √ | |
| 442411 | [Glepidotin B](https://old.tcmsp-e.com/molecule.php?qn=4829) | high | √ | √ | √ | √ | √ | |
| 5317768 | [Glypallichalcone](https://old.tcmsp-e.com/molecule.php?qn=4835) | high | √ | √ | √ | √ | √ | |
| 5318999 | [Licochalcone B](https://old.tcmsp-e.com/molecule.php?qn=4841) | high | √ | √ | √ | √ | √ | |
| 49856081 | [licochalcone G](https://old.tcmsp-e.com/molecule.php?qn=4848) | high | √ | √ | √ | √ |  | |
| 392443 | [licoisoflavanone](https://old.tcmsp-e.com/molecule.php?qn=4885) | high | √ | √ | √ | √ | √ | |
| 10336244 | [shinpterocarpin](https://old.tcmsp-e.com/molecule.php?qn=4891) | high | √ | √ | √ | √ | √ | |
| 11267805 | [(E)-3-[3,4-dihydroxy-5-(3-methylbut-2-enyl)phenyl]-1-(2,4-dihydroxyphenyl)prop-2-en-1-one](https://old.tcmsp-e.com/molecule.php?qn=4898) | high | √ | √ | √ | √ | √ | |
| 124052 | [Glabridin](https://old.tcmsp-e.com/molecule.php?qn=4908) | high | √ | √ | √ | √ | √ | |
| 124049 | [Glabranin](https://old.tcmsp-e.com/molecule.php?qn=4910) | high | √ | √ | √ | √ | √ | |
| 480774 | [Glabrene](https://old.tcmsp-e.com/molecule.php?qn=4911) | high | √ | √ | √ | √ | √ | |
| 5317652 | [Glabrone](https://old.tcmsp-e.com/molecule.php?qn=4912) | high | √ | √ | √ | √ | √ | |
| 5317300 | [Eurycarpin A](https://old.tcmsp-e.com/molecule.php?qn=4915) | high | √ | √ | √ | √ | √ | |
| 23724664 | [(-)-Medicocarpin](https://old.tcmsp-e.com/molecule.php?qn=4924) | high | √ | √ | √ | √ | √ | |
| 480873 | [1-Methoxyphaseollidin](https://old.tcmsp-e.com/molecule.php?qn=4959) | high | √ | √ | √ | √ | √ | |
| 15228662 | [3'-Hydroxy-4'-O-Methylglabridin](https://old.tcmsp-e.com/molecule.php?qn=4966) | high | √ | √ | √ | √ | √ | |
| 5318998 | [licochalcone a](https://old.tcmsp-e.com/molecule.php?qn=497) | high | √ | √ | √ | √ | √ | |
| 101666840 | [Kanzonol F](https://old.tcmsp-e.com/molecule.php?qn=4988) | high | √ | √ | √ | √ |  | |
| 25015742 | [7,2',4'-trihydroxy－5-methoxy-3－arylcoumarin](https://old.tcmsp-e.com/molecule.php?qn=4990) | high | √ | √ | √ | √ | √ | |
| 268208 | [7-Acetoxy-2-methylisoflavone](https://old.tcmsp-e.com/molecule.php?qn=4991) | high | √ | √ | √ | √ | √ | |
| 92503 | [Vestitol](https://old.tcmsp-e.com/molecule.php?qn=500) | high | √ | √ | √ | √ | √ | |
| 480780 | [Gancaonin G](https://old.tcmsp-e.com/molecule.php?qn=5000) | high | √ | √ | √ | √ | √ | |
| 5481949 | [Gancaonin H](https://old.tcmsp-e.com/molecule.php?qn=5001) | high | √ | √ | √ | √ |  | |
| 15840593 | [Licoagrocarpin](https://old.tcmsp-e.com/molecule.php?qn=5003) | high | √ | √ | √ | √ | √ | |
| 5280343 | [quercetin](https://old.tcmsp-e.com/molecule.php?qn=98) | high | √ | √ | √ | √ | √ | |
| 323 | Coumarin | high | √ |  | √ | √ |  | |
| 444539 | Cinnamic Acid | high | √ |  | √ | √ |  | |
| 72 | 3,4-Dihydroxybenzoic Acid | high | √ |  | √ | √ |  | |
| 637511 | Cinnamaldehyde | high | √ |  | √ | √ |  | |
| [131752069](https://pubchem.ncbi.nlm.nih.gov/compound/131752069) | Anhydrocinnzeylanine | high | √ | √ | √ | √ | √ | |
| [73099741](https://pubchem.ncbi.nlm.nih.gov/compound/73099741) | Anhydrocinnzeylanol | high | √ | √ | √ | √ | √ | |
| 5318169 | 2'-Hydroxycinnamaldehyde | high | √ |  | √ | √ |  | |
| 873 | Melilotic Acid | high | √ | √ | √ | √ |  | |
| 641298 | 2-Methoxycinnamaldehyde | high | √ | √ | √ | √ |  | |
| [439533](https://pubchem.ncbi.nlm.nih.gov/compound/439533) | [(-)-taxifolin](https://old.tcmsp-e.com/molecule.php?qn=1736) | high | √ | √ | √ | √ | √ | |
| 9064 | [(+)-catechin](https://old.tcmsp-e.com/molecule.php?qn=492) | high | √ | √ | √ | √ | √ | |
| [182232](https://pubchem.ncbi.nlm.nih.gov/compound/182232) | [ent-Epicatechin](https://old.tcmsp-e.com/molecule.php?qn=73) | high | √ | √ | √ | √ | √ | |
| 439533 | [taxifolin](https://old.tcmsp-e.com/molecule.php?qn=4576) | high | √ | √ | √ | √ | √ | |
| 5351516 | [Peroxyergosterol](https://old.tcmsp-e.com/molecule.php?qn=11169) | high | √ |  | √ | √ |  | |
| 21594250 | machaerinic acid lactone | high | √ |  | √ | √ |  | |
| 5281646 | macluraxanthone | high | √ | √ | √ | √ |  | |
| 439260 | noradrenaline | high | √ | √ | √ | √ |  | |
| 6999736 | norarmepavine | high | √ | √ | √ | √ | √ | |
| 54670067 | vitamin c | high | √ |  | √ | √ |  | |
| 12305768 | Alphitolic Acid | high | √ |  | √ | √ |  | |
| 122691 | Lysicamine | high | √ | √ | √ | √ | √ | |
| 197017 | N-Methylasimilobine | high | √ | √ | √ | √ | √ | |
| 23335 | Caaverine | high | √ | √ | √ | √ | √ | |
| 15515703 | Jujubogenin | high | √ |  | √ | √ |  | |
| 3085285 | Juzirine | high | √ | √ | √ | √ | √ | |
| 14729078 | Sanjoinenine | high | √ |  | √ | √ |  | |
| 14729076 | Sanjoinine B | high | √ |  | √ | √ | √ | |
| 44566617 | Sanjoinine D | high | √ |  | √ | √ | √ | |
| 14729081 | Sanjoinine F | high | √ |  | √ | √ | √ | |
| 10460277 | Sanjoinine G1 | high | √ |  | √ | √ | √ | |
| 160487 | [(S)-Coclaurine](https://old.tcmsp-e.com/molecule.php?qn=1522) | high | √ | √ | √ | √ | √ | |
| 102063083 | [zizyphusine](https://old.tcmsp-e.com/molecule.php?qn=1546) | high | √ | √ | √ | √ | √ | |
| 15693541 | 1,2,3,6,7-pentamethoxyxanthone | high | √ | √ | √ | √ | √ | |
| 5316765 | 1,3-dihydroxy-4,5-dimethoxyxanthone | high | √ | √ | √ | √ | √ | |
| 5316837 | 1,6-dihydroxy-3,5,7-trimethoxyxanthone | high | √ | √ | √ | √ | √ | |
| 5316766 | 1,6-Dihydroxy-3,7-dimethoxyxanthone | high | √ | √ | √ | √ | √ | |
| 15267570 | 1,7-dimethoxyxanthone | high | √ | √ | √ | √ | √ | |
| 11701473 | 1-carboethoxy-beta-carboline | high | √ | √ | √ | √ | √ | |
| 5488808 | 1-hydroxy-3,7-dimethoxyxanthone | high | √ | √ | √ | √ | √ | |
| 442290 | 1-Peroxyferolide | high | √ | √ | √ | √ | √ | |
| 735755 | 3,4,5-TRIMETHOXYCINNAMIC ACID | high | √ | √ | √ | √ | √ | |
| 717531 | 3,4-dimethoxy cinnamic acid | high | √ | √ | √ | √ | √ | |
| 148724 | 5,6,7-Trimethoxycoumarin | high | √ | √ | √ | √ | √ | |
| 71378875 | 6-hydroxy-1,2,3,7-tetramethoxyxanthone | high | √ | √ | √ | √ | √ | |
| 148657 | aristolactam a | high | √ | √ | √ | √ | √ | |
| 243 | benzoic acid | high | √ |  | √ | √ |  | |
| 5375436 | beta-carboline-1-propionic acid | high | √ | √ | √ | √ | √ | |
| 10364 | carvacrol | high | √ |  | √ | √ |  | |
| 638011 | citral | high | √ |  | √ | √ |  | |
| 7794 | citronellal | high | √ |  | √ | √ |  | |
| 15460049 | cordarine | high | √ |  | √ | √ | √ | |
| 5281618 | geraldone | high | √ | √ | √ | √ | √ | |
| 5281404 | Harman | high | √ | √ | √ | √ |  | |
| 5280953 | harmine | high | √ | √ | √ | √ | √ | |
| 8194 | laurinaldehyde | high | √ | √ | √ | √ |  | |
| 5281167 | leaf alcohol | high | √ |  | √ | √ |  | |
| 6549 | linalool | high | √ |  | √ | √ |  | |
| 189168 | norcepharadione b | high | √ | √ | √ | √ | √ | |
| 64961 | Norharman | high | √ | √ | √ | √ |  | |
| 64696 | Norhyoscyamine | high | √ | √ | √ | √ | √ | |
| 160179 | Perlolyrine | high | √ | √ | √ | √ | √ | |
| 3081016 | piperolactam a | high | √ | √ | √ | √ | √ | |
| 23618202 | s-(2-carboxyethyl)-l-cysteine | high | √ | √ | √ | √ |  | |
| 227830 | tenulin | high | √ | √ | √ | √ | √ | |
| 6989 | thymol | high | √ |  | √ | √ |  | |
