# Supplementary figures and images for: Integrated network pharmacology, molecular docking and experimental validation to explore the mechanism of Dingji Fumai Decoction against LQTS
Source: Sci Rep. 2025 Jul 2;15:23037. doi: 10.1038/s41598-025-06515-7 (PMC12215522; doi:10.1038/s41598-025-06515-7)

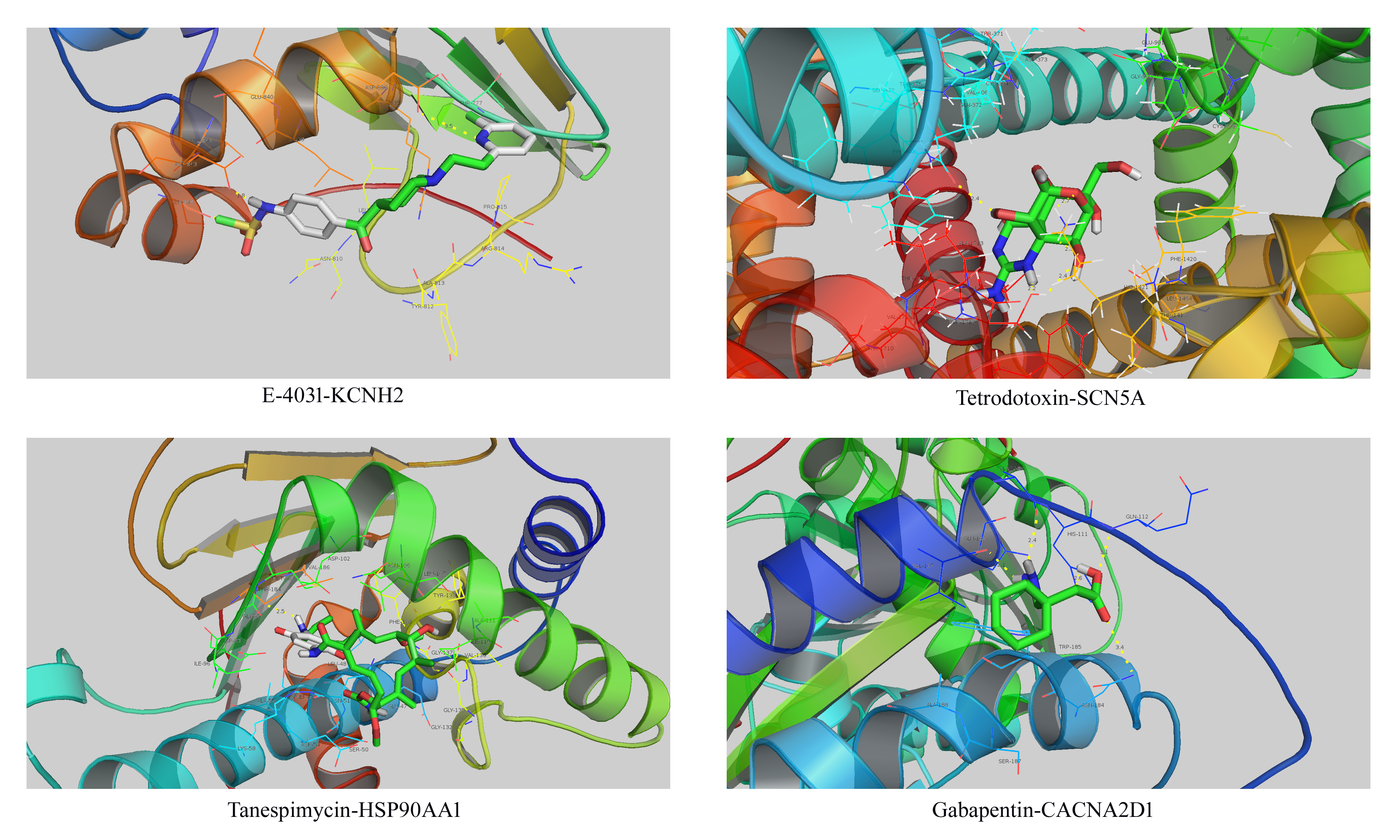

Supplement: Supplementary file 2 — Supplementary Material 2 [file 41598_2025_6515_MOESM2_ESM.tif]
